# Genetic Basis of the Negative Response to the Use of Triptans for the Treatment of Migraine—A Systematic Review and Meta‐Analysis

**DOI:** 10.1002/brb3.70967

**Published:** 2025-10-21

**Authors:** Victoria Gomes Andreata, Ruan Pablo Duarte Freitas, Jéssica da Silva Nascimento, Patrícia Sodré Araújo, Felipe Eustáquio dos Santos Guedes, João Vítor Benjamim Pires, Narel Moita Carneiro Nogueira Falcão, Natasha da Silva Leitão, Alcylene Carla de Jesus dos Santos, Ana Patrícia Pascoal Queiroz, Astria Ferrão Dias Gonzales, Maica Matos Leão, Emília Katiane Embiruçu de Araújo Leão, Juliana Côrtes Freitas, Acássia Benjamim Leal Pires

**Affiliations:** ^1^ Hospital Municipal Doutor Waldemar Tebaldi São Paulo Brazil; ^2^ Faculdade ZARNS Bahia Brazil; ^3^ Centro Universitário Dom Pedro II, UNIDOMPEDRO/AFYA Salvador Bahia Brazil; ^4^ Universidade Federal da Bahia (UFBA) Salvador Bahia Brazil; ^5^ Hospital Macrorregional de Caxias Dr. Everaldo Aragão Maranhão Brazil; ^6^ Grupo da Dor Orofacial do Amazonas (DOFAM) Manaus Amazonas Brazil; ^7^ Universidade do Estado da Bahia, DCV, UNEB Salvador Bahia Brazil; ^8^ Programa de Pós‐Graduação em Saúde Coletiva (PPGSC), UNEB Salvador Bahia Brazil; ^9^ Programa De Pós‐Graduação em Ciências Farmacêuticas (PGFARMA), UNEB Salvador Bahia Brazil

**Keywords:** genetic polymorphisms, migraine disorders, pharmacogenetics, tryptamines

## Abstract

**Introduction:**

Migraine is a widespread and disabling neurological disorder, and triptans are a primary treatment for acute episodes. However, around 40% of patients are non‐responsive. Genetic polymorphisms can influence drug effectiveness, and several association studies exist. This systematic review consolidates findings from etiological studies, which may provide greater certainty about their use in predicting the risk of low response to triptans.

**Method:**

This study followed COSMOS‐E guidelines and employed databases like PubMed, Web of Science, and Embase until 2023. The Newcastle–Ottawa Scale (NOS) was used to assess quality, and statistical meta‐analysis was performed.

**Results:**

A comprehensive literature search identified 1421 articles from which 30 met eligibility for full‐text review, and 9 studies were included in the final analysis. Although the overall analysis did not confirm a statistically significant association, subgroup analyses demonstrated significant relationships between SLC6A4, 5‐HT1B, and COMT polymorphisms and triptan nonresponse, while CALCA and PRDM16 had moderate evidence, and GRIA1 and SCN1A polymorphisms exhibited limited evidence for non‐response. While our analysis revealed that genetic polymorphisms are an essential cause of heterogeneity in response to triptans, the included studies showed substantial variability and methodological inconsistency. Polygenic risk scores (PRS) and combined genetic approaches, including prospective clinical trials and multi‑omics integration, can help stratify triptan nonresponders and inform decision‑making in precision and personalized medicine (PPM).

**Conclusions:**

These results underscore the potential utility of these genetic associations in tailored migraine therapy and reinforce the involvement of serotonergic and dopaminergic pathways in triptan efficacy. Future studies with larger and more homogeneous cohorts are essential to validate these associations. Still, clinical implementation of polygenic risk score (PRS) may offer a more effective pathway for applying PPM in migraine care.

## Graphical abstract

To systematize the genetic basis correctly associated with nonresponse to triptan, we revised etiological studies on Web of Science, Embase, and PubMed until 2023. We found that only polymorphisms in the SLC6A4, 5‐HT1B, and COMT genes have a stronger association with this condition. More robust studies are needed to increase this certainty.

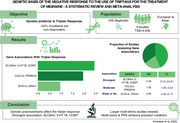

## 1 Introduction

Migraine is a common disabling primary headache disorder distinguished from other primary headaches by its complex clinical presentation and variability of associated symptoms (Puledda et al. 2024). Recognized by the World Health Organization as the most burdensome neurological disorder in terms of societal impact and the sixth leading cause of disability worldwide, migraine significantly affects patients’ quality of life, family relationships, workplace output, education, and economic well‐being (Feigin et al. 2021). It remains a major cause of disability worldwide, and care still benefits from more individualized acute and preventive strategies, including attention to pharmacogenetic variability (Raggi et al. 2024; Pomes et al. 2019).

Global prevalence of migraine is estimated at approximately 15%, accounting for about 4.9% of total years lived with disability (YLDs) worldwide (Stovner et al., 2018). In Latin America, prevalence is also approximately 15% (Pacheco‐Barrios et al., 2023). In primary care, the EMR France‐Mig study estimated that 26.7% of adults with migraine met criteria for triptan failure: 23.8% were ineligible (including 8.1% with cardiovascular contraindications and 12.2% due to age ≥ 65 years) and 3.6% were resistant, quantifying how often non‐response or ineligibility is encountered in routine care (Lanteri‐Minet et al. 2025)

Migraine can be classified into different subtypes, mainly migraine without aura (MO) and migraine with aura (MA), and can be episodic or chronic (Goadsby and Evers 2020; Cammarota et al. 2024). MO is a recurrent headache disorder manifesting in attacks that last 4–72 h, characterized by intense, recurring unilateral pain accompanied or not by nausea, vomiting, photophobia, phonophobia, and, in some cases, motion sensitivity. MA is distinct as it follows a cyclic course of four phases: the premonitory phase, the transient neurological symptoms known as aura, the headache phase, and the postdrome (Goadsby and Evers 2020). Chronic migraine is defined by the occurrence of headache on 15 or more days per month for 3 months or more. It is associated with greater functional impairment and reduced quality of life (Hovaguimian and Roth 2022).

Migraine headache arises from neurogenic inflammation of first‐division trigeminal sensory neurons innervating cerebral blood vessels and meninges. These neurons are activated by cortical spreading depression (CSD), a slowly propagating wave of neuronal depolarization across the cerebral cortex. Pro‐inflammatory mediators such as calcitonin gene‐related peptide (CGRP) and substance P (SP) lead to stimulation of small‐diameter, unmyelinated C‐fibers responsible for pain transmission (Charles 2018). These trigeminal afferents are modulated by serotonin (5‐hydroxytryptamine, 5‐HT) receptors (Vila‐Pueyo 2018).

Genetic factors influence migraine susceptibility, with heritability estimated at around 42%, indicating polygenic inheritance with strong environmental modulation; migraine with aura shows even greater genetic predisposition (De Boer et al. 2020; Grangeon et al., 2023). Familial and sporadic hemiplegic migraine (HM) are monogenic but rare (prevalence ≈0.003%), with causal mutations identified in CACNA1A, ATP1A2, and SCN1A, although sporadic occurrence suggests additional genetic contributors (Alfayyadh et al. 2024).

Polygenic migraine arises from the cumulative effect of multiple low‐impact variants. An extensive case‐control GWAS study identified 123 migraine risk loci: nine associated with overall susceptibility, two (near SPINK2 and FECH) associated with MO, and three (HMOX2, CACNA1A, MPPED2) with MA (Hautakangas et al. 2022). In a multi‐ethnic GWAS, 22 risk loci were found, 10 novel and three explicitly associated with migraine in females (CPS1 rs1047891, PBRM1 rs11718509, SLC25A21 rs10150336) (Choquet et al. 2021). Alongside genetic variants, epigenetic influences are under investigation: current insights point to microRNA profiling, DNA methylation, and histone modifications as potential biomarkers for migraine (Gallardo et al. 2023)

Among therapeutic options, the drugs classified as triptans stand out as selective serotonergic agonists acting directly on 5‐HT1B and 5‐HT1D receptors, demonstrating high efficacy in treating migraine attacks. Its use is considered a prophylactic measure(Cameron et al. 2015; Dodick 2018). By activating these receptors, triptans induce vasoconstriction of cranial arteries and block pro‐inflammatory releases, alleviating pain and other symptoms such as nausea, photophobia, and allodynia (i.e., perception of neurological pain due to a normally non‐painful stimulus, such as thermal or mechanical stimuli) (Goadsby and Evers 2020, Cargnin et al. 2015, Pijpers et al. 2023).

Nowadays, the U.S. Food and Drug Administration (FDA) includes a total of seven triptans designed explicitly for the immediate relief of acute migraine episodes, such as sumatriptan, eletriptan, naratriptan, zolmitriptan, rizatriptan, frovatriptan, and almotriptan (Sacco and Kurth, 2014). In Brazil, migraine management follows guidelines from the Brazilian Headache Society. At the same time, the Ministry of Health adheres mostly to FDA recommendations for acute treatment (Nacazume and Marcourakis, 2019). There are currently four drugs of this class on the market in Brazil: sumatriptan, naratriptan, rizatriptan, and zolmitriptan. The Brazilian Headache Society also suggests a mini‐prophylaxis approach combining hormonal agents, anti‐inflammatory drugs, and triptans (Melhado et al. 2022).

However, the principal safety concern with triptans is still related to their ability to cause coronary or peripheral arterial constriction that could result in serious adverse cardiac or peripheral vascular events (Liu et al. 2024). These clinical limitations, together with safety concerns, have influenced regulatory decisions regarding triptan use (Cameron et al. 2015, Ferrari et al. 2001). As a result, the FDA has adopted certain standard or class labeling for triptans and warned that overuse of acute migraine drugs (e.g., ergotamine, triptans, opioids, or a combination of medications for 10 or more days per month) may lead to exacerbation of headache (Goadsby et al. 2017).

In fact, migraine is associated with a modest increase in long‐term atherosclerotic cardiovascular disease risk (HR 1.12, 95% CI 1.05–1.20), but a UK Biobank analysis found no significant association between acute treatments, including triptans, and incident ASCVD events (Huang et al. 2025). In a large cross‐sectional cohort of 1047 referred patients, clinicians in primary or secondary care had advised a triptan in 63% of cases, which reflects their ongoing central role in acute management (Fitzek et al. 2025).

Besides specificity and efficacy, some migraine patients are not eligible for triptan treatment, and some are triptan non‐responders or show essential side effects. Only 60% of triptan users report sustained 24‐h pain relief, and with ergotamine, this rate falls to 8%. The patient is considered a non‐responder when there is no headache improvement in 2 h or recurrence after initial pain relief. No triptan achieves a 100% response rate; 30%–60% of patients do not experience headache relief within 2 h with certain triptans, and recurrence occurred in 20%–40% (De Boer et al. 2020, Ruscheweyh et al. 2023). Using European Headache Federation criteria, registry data indicate that 13.1% of patients are triptan‐resistant (failure of ≥ 2 triptans) and 0.6% are triptan‐refractory (≥ 3), providing a clear target phenotype for genetic studies (Ruscheweyh et al. 2023).

Many factors may influence triptan's response, such as the right timing (better response when taken upon the appearance of the first symptom), comorbidities, concomitant usage of other drugs, problems in absorption, and individual variations in pharmacokinetics (De Boer et al. 2020). Poor responses to triptans are also associated with severity and chronicity (Ruscheweyh et al. 2023). Switching to other triptans is common, though overuse remains a challenge.

Nonetheless, genetic factors are being considered when evaluating this response. For instance, a variant of the gene COMT rs4680 (Val158Met) was shown to reduce COMT's enzymatic activity and elevate extracellular levels of dopamine and norepinephrine (Tunbridge 2010). Individuals carrying the allele MET showed higher sensitivity to pain due to reduced depuration of catecholaminergic neurotransmitters (Ferreira Do Couto et al. 2024). COMT (Catechol‐*O*‐Methyltransferase), alongside MAO‐A (Monamine Oxidase A), the main enzymes involved in tyrosine metabolism, were also significantly increased in migraine model rats (Zhu et al. 2022). The role of the dopaminergic system in migraine, including vascular regulation and blood‐brain barrier permeability, corroborated these findings, despite the small cohort, and highlighted DRD2/NcoI as a potential pharmacogenetic predictor of response to rizatriptan (Zhang et al. 2022; Asuni et al. 2007).

Determining to what extent genetics affects the treatment could avoid triptan prescription to patients likely to be non‐responders. Recent discoveries in human genetics and consequent advances in genetic testing may give rise to new reflections on drug therapies for diseases, resulting in personalized treatments (Hussen et al. 2022). The genotype defines the phenotypic manifestations of diseases under certain conditions, underscoring the value of investigating response variability in migraine (Dickmann and Ware 2016). Thus, genetic alterations in the genes could be related to the different pharmacological responses perceived in the drug treatment of migraine.

This systematic review aims to explore the evidence of the genetic basis for the negative response to triptans for migraine treatment. Key polymorphisms available in etiological studies were screened to provide insights into the likely role of genetic risk factors in therapeutic outcomes, emphasizing the importance of individualized treatment approaches.

## 2 Methodology

### 2.1 Study Design

This study followed a systematic review and meta‐analysis approach, focusing on case‐control and cohort studies to investigate genetic factors affecting triptan response in migraine treatment. This study aims to systematically synthesize evidence on genetic predispositions influencing triptan efficacy using peer‐reviewed, indexed studies. The review adheres to COSMOS‐E guidelines to ensure rigor in study selection and data analysis. Before conducting this review, the protocol was registered in PROSPERO (CRD2024598554) to ensure methodological transparency. The PRISMA checklist used in this systematic review is in the supplementary material (Table S1).

### 2.2 Search Strategy

A systematic search strategy was implemented to identify relevant studies in three primary databases: PubMed, Web of Science, and Embase. Search terms incorporated Boolean operators and included key terms such as “migraine,” “triptans,” and “genetics” (Table S2). Open‐access and subscription‐based studies were included, and authors of relevant studies were contacted for additional data where necessary.

### 2.3 Eligibility Criteria

The eligibility criteria were observational studies (case‐control and cohort designs) in adult patients (≥ 18 years) with a diagnosis of migraine according to the International Headache Society (IHS) criteria (2018). Low triptan response was defined as the persistence of migraine symptoms with recommended dosages.

Genetic factors associated with triptan response were assessed, including variations influencing metabolism, absorption, receptor binding, and adverse effects. There was no restriction on gender or ethnicity.

### 2.4 Data Extraction

Two independent reviewers extracted data and resolved discrepancies by achieving a consensus or referring the issue to a third reviewer. The extracted data included study characteristics, participant demographics, genetic variants studied, and key outcomes related to triptan response. Confounding variables like age, gender, and comorbidities. We also collected statistics, such as effect estimates in the form of odds ratios (ORs) or risk ratios with their standard errors, for the statistical analysis.

### 2.5 Quality and Risk of Bias Assessment

The quality and risk of bias of the observational studies were assessed using the Newcastle–Ottawa Scale (NOS). The NOS assessed studies based on three domains: selection (population representativeness), Comparability (control for confounders such as age and comorbidities), and Exposure (assessment of genetic variants and follow‐up quality).

We only analyzed research with a score of 5 or higher since they were categorized as moderate to high risk, and those below or equal to 4 were excluded because of high bias risk. Where there was a scoring disagreement, the papers’ authors were discussed with a third reviewer.

### 2.6 Outcome

This study aimed to determine the genetic factors that affect triptan efficacy. It considers variants that affect drug metabolism, absorption, receptor binding efficiency, and possible side effects.

The analysis included the following triptans: almotriptan, eletriptan, frovatriptan, naratriptan, rizatriptan, sumatriptan, and zolmitriptan. These drugs were included based on their approval for migraine treatment by major regulatory agencies, including the British National Formulary (BNF, UK), European Medicines Agency (EMA, Europe), US FDA, and equivalent authorities in Germany (BÉArM), France (ANSM), Japan (PMDA), Australia (TGA), and Brazil (ANVISA).

### 2.7 Statistical Analysis

A meta‐analysis was performed to quantitatively combine the data from the selected research and determine the overall effect of genetic polymorphisms on triptan response. Due to the high heterogeneity of the studies, a random effects model was used to compare across studies. The Mantel–Haenszel method calculated the pooled ORs with their respective 95% confidence intervals (CI). Heterogeneity was evaluated through Cochran's *Q*‐test and the *I* ^2^ statistic and was considered significant for substantial heterogeneity when the *I* ^2^ value was greater than 75%. Between‐study variance was quantified using the *τ* ^2^ statistic.

Subgroup analyses were carried out to identify possible sources of heterogeneity by dividing genetic variants into neurotransmitter‐related, ion channel‐related, and migraine‐specific genes. The leave‐one‐out sensitivity analysis was performed to determine each study's effect on the overall effect sizes. The funnel plot and Egger's test were used to assess publication bias; if asymmetry was found, the Trim‐and‐Fill method was used to adjust for possible bias. Meta‐regression analysis checked whether publication year and the number of polymorphisms influenced effect sizes.

All statistical analyses were performed using Review Manager (RevMan) V. 8.16.0 and the RCore Team. R: A Language and Environment for Statistical V. 4.4.2. Vienna to ensure standardized and reproducible results.

### 2.8 Declaration of Artificial Intelligence Generated Content

We confirm that ChatGPT was used exclusively for grammar revision and improvement of English language fluency. AI generated no scientific, methodological, or interpretative content.

## 3 Results

### 3.1 Characteristics of Identified Studies

**FIGURE 1:**
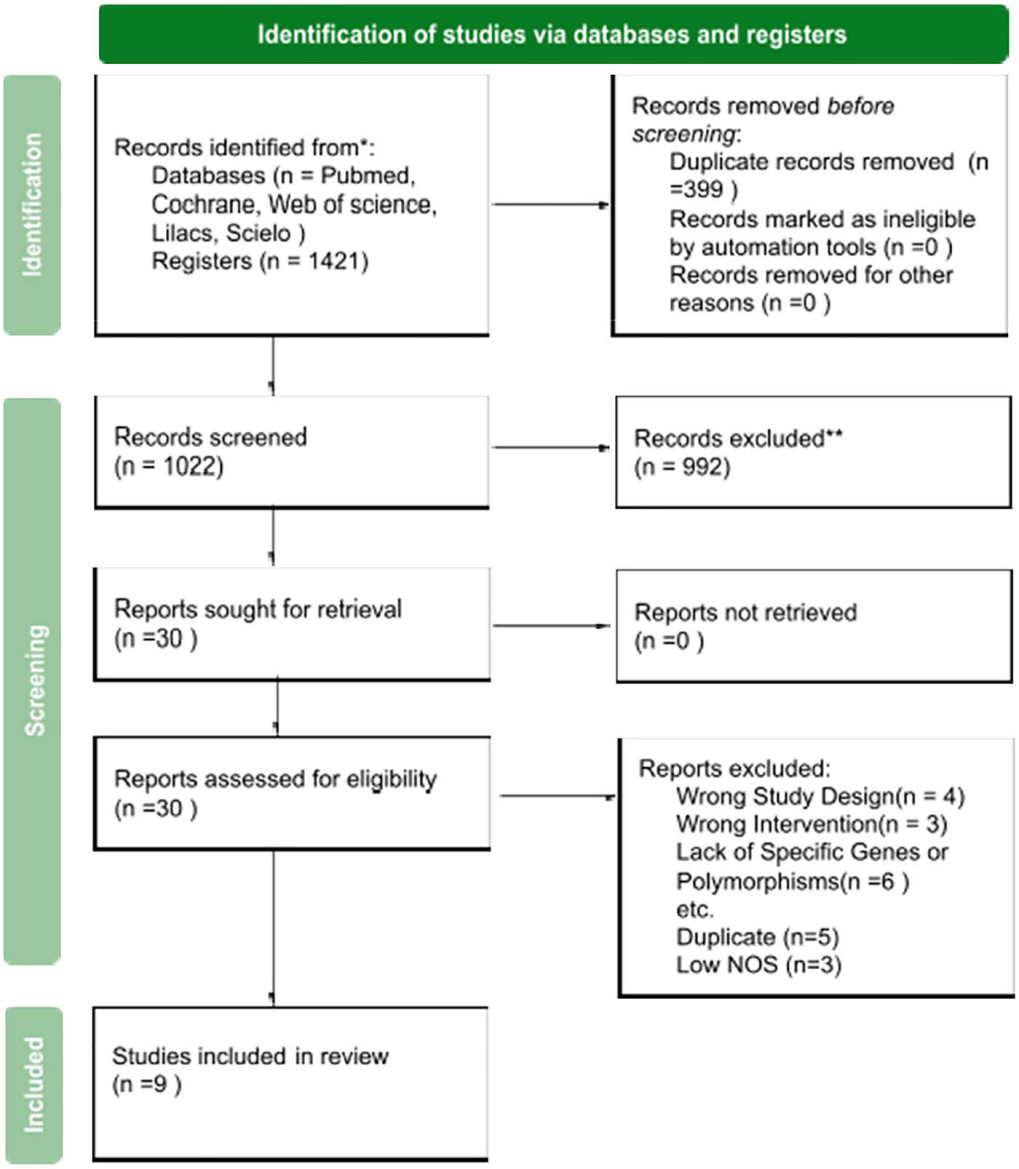
PRISMA 2020 Flow Diagram for Study Selection. Systematic representation of the identification, screening, eligibility assessment, and inclusion of studies in the meta‐analysis. The diagram illustrates the number of records retrieved from databases and registers, the number of excluded studies at each stage, and the final count of studies included in the systematic review.

A comprehensive literature search identified 1421 articles from which 30 were eligible for full‐text review, and 9 studies were included in the final analysis (Cargnin et al. 2015; MaassenVanDenBrink et al. 1998, Gentile et al. 2010; Cargnin et al. 2013; Terrazzino et al. 2010; Cargnin et al. 2019; Ishii et al. 2012; Cargnin et al. 2014; Christensen et al. 2016). The excluded reports were: wrong study design (*n* = 4), wrong intervention (*n* = 3), Lack of specific genes or polymorphisms (*n* = 6), duplicates (*n* = 5), and low NOS (*n* = 3). These studies investigated the influence of genetic polymorphisms on the clinical response to triptans in treating migraines (Figure 1).

The quality of the included studies was evaluated using the Newcastle–Ottawa Scale (NOS), which assesses selection, comparability, and outcome/exposure domains. Studies scoring ≥ 5 points were considered moderate to high quality and included in the final analysis, while those scoring ≤ 4 points were excluded due to a high risk of bias (Table S3).

The main characteristics of the identified studies are summarized (Table 1). These studies were conducted across various geographic regions, including Europe (Italy, the Netherlands, Denmark) and Asia (Japan). The studies included populations ranging from 35 participants (Gentile et al. 2010) to 2198 participants (Singh et al. 2020). Participant ages ranged from 18 to 81 years, with a predominance of female participants, including 79% (Cargnin et al. 2013). The methodologies employed included case‐control studies (Cargnin et al. 2015; MaassenVanDenBrink et al. 1998; Terrazzino et al. 2010), prospective cohort studies (Cargnin et al. 2013; Terrazzino et al. 2010), and retrospective observational designs (Nacazume and Marcourakis, 2019). Most studies focused on evaluating single‐nucleotide polymorphisms (SNPs) or haplotypes in genes associated with serotonergic, dopaminergic, or metabolic pathways.

**TABLE 1:** Characteristics of included studies. This table summarizes the included studies investigating the genetic influence on triptan response. Study arms differentiate experimental (E) and control (C) groups when applicable, while gender is consistently represented as Males/Females. Age data is presented in the format mean (±SD) or range, and the triptan medications administered in each study.

Study	Country	Type of study	Population	Study arms	Gender (M/F)	Mean age (±SD or range)	Types of triptans
MaassenVanDenBrink et al. (1998)	Netherlands	Case‐control	66	E: 40 migraine, C: 26 control	0/35 (E), 26/0 (C)	47 (20–69) (E), 35 (C)	Sumatriptan
Gentile et al. (2010)	Italy	Cohort	150	No control group	0/124	25–81	Sumatriptan, Eletriptan, Rizatriptan, Almotriptan, Frovatriptan, Zolmitriptan
Terrazzino et al. (2010)	Italy	Cohort	130	No control group	0/102	37.6 (±10.3)	Eletriptan, Rizatriptan, Sumatriptan, Frovatriptan, Almotriptan, Zolmitriptan
Ishii et al. (2012)	Japan	Cross‐sectional	60	No control group	0/46	44.1 (±10.9)	Sumatriptan, Zolmitriptan, Eletriptan, Rizatriptan, Naratriptan
Cargnin et al. (2013)	Italy	Cohort	198	No control group	0/63	40.9 (±11.3)	Rizatriptan, Eletriptan, Almotriptan, Sumatriptan, Zolmitriptan, frovatiptan
Cargnin et al. (2014)	Italy	Case‐control	498	E: 186 migraine without aura, C: 312 control	21/165 (E), 116/196 (C)	39 (±10.6) (E), 56 (±18.1) (C)	Frovatriptan, Rizatriptan, Eletriptan, Almotriptan, Sumatriptan, Zolmitriptan
Cargnin et al. (2015)	Italy	Case‐control	558	EMSA: 219, CUEM: 130, C: 209	44/175 (EMSA), 32/98 (CUEM), 51/158 (C)	38.4 (±10.6) (EMSA), 47.8 (±11.1) (CUEM), 50.0 (±15.5) (C)	Frovatriptan, Eletriptan, Rizatriptan, Almotriptan, Sumatriptan, Zolmitriptan
Christensen et al. (2016)	Denmark	Cohort	2198	Discovery: 1806, Replication: 392	1 M/4.6F (D), 1 M/6F (R)	44.7 (D), 42.9 (R)	Triptans as 5HT1B/D receptor selective agonists for acute migraine treatment
Cargnin et al. (2019)	Italy	Cohort	172	No control group	19/153	38.7 (±11)	Sumatriptan, Eletriptan, Almotriptan, Zolmitriptan, Rizatriptan, Frovatriptan

### 3.2 Genetic Variation Associations with Triptan Response

As detailed in Table 2, variations in serotonin‐related genes were a primary focus of the included studies. The G861C variant in the HTR1B gene was investigated. Still, the study did not identify significant associations between this variant and clinical response to triptans, including sumatriptan (MaassenVanDenBrink et al. 1998). In contrast, the STin2 VNTR variant in the serotonin transporter gene SLC6A4 demonstrated a significant association (Terrazzino et al. 2010). Specifically, patients with the STin2 12/12 genotype showed a higher likelihood of inconsistent responses to triptans, indicating that this genetic variant may contribute to variability in treatment outcomes.

**TABLE 2:** Study characteristics and genetic variants analyzed in relation to triptan response. The table presents the specific genes, polymorphisms, and their associated outcomes, along with effect sizes (odds ratios or relative risks) and corresponding confidence intervals extracted from meta‐analysis and individual study findings.

Study	Gene	Polymorphism	Outcome	Effect Size (OR/RR)	Confidence Interval (CI)
MaassenVanDenBrink et al. (1998)	5‐HT1B Receptor	G861C, T‐267G	Clinical response to sumatriptan	Not Significant	N/A
Terrazzino et al. (2010)	SLC6A4, GNB3, DRD2	STin2 VNTR, 5‐HTTLPR; C825T; NcoI, TaqI A	Triptan treatment efficacy	Not Significant	N/A
Gentile et al. (2010)	CYP1A2	−163A>C	Risk of inconsistent response to triptans	3,363	(1.262–8.966)
Ishii et al. (2012)	5−HTTLPR, 5‐HTTVNTR, 5‐HT2A, TNF‐b, GNB3	s/s, s/l, l/l, s/xl; 12/12, 12/10, 12/9, 10/10; T102C; G252A (rs909253); C825T	Predictors of inconsistent response to triptans	14,085	(1.253–166.667)
Cargnin et al. (2013)	COMT	Val158Met (rs4680)	Risk of poor response to triptans	0.21	(0.04–0.98)
Cargnin et al. (2014)	GRIA1	rs548294, rs2195450	Association with migraine without aura and triptan response	0.89 (rs548294), 0.93 (rs2195450)	(0.60–1.34), (0.62–1.39)
Cargnin et al. (2015)	CALCA, RAMP1	rs3781719; rs7590387, rs3754701	Risk of migraine transformation to medication overuse headache	0.49	(0.28‐0.86)
Christensen et al. (2016)	PRDM16, LRP1, TRPM8, MMP16, PHACTR1, TSPAN2, FHL5, MEF2D, ASTN2, TGFBR2, AJAP1, c7orf10	rs2651899 (PRDM16), rs11172113 (LRP1), rs7577262 (TRPM8), rs10504861 (MMP16), rs9349379 (PHACTR1), rs12134493 (TSPAN2), rs13208321 (FHL5), rs2274316 (MEF2D), rs6478241 (ASTN2), rs6790925 (TGFBR2), rs10915437 (AJAP1), rs4379368 (c7orf10)	Triptan and ergotamine treatment response. Combined genetic effect on triptan and ergotamine response	1.69	(1.26‐2.28)
Cargnin et al. (2019)	PRDM16, MMP16, LRP1, FGF6, TRPM8	rs9349379; rs2078371; rs6478241, rs11172113; rs1024905; rs6724624	Association with inconsistent triptan response. Combined genetic effect on inconsistent triptan response	0.62	(0.43‐0.89)

The influence of dopaminergic system genes was also thoroughly examined. The DRD2 rs6275 variant was associated with improved rizatriptan response and confirmed in subsequent studies (Terrazzino et al. 2010; Ishii et al. 2012). Conversely, multiple studies consistently associated the rs4680 (Val158Met) variant in the COMT gene with triptan response. Met/Met carriers showed poorer frovatriptan response, confirmed in later studies (Cargnin et al. 2013, 2015).

### 3.3 Multi‐Locus Models and Additional Genes

Multi‐locus genetic models were explored to predict triptan efficacy. Cargnin et al. (2015) introduced a genetic risk score (GRS) incorporating SNPs such as TRPM8 rs6724624 and FGF6 rs1024905, demonstrating an inverse association with inconsistent responses to triptans. This approach underscored the potential of combining genetic markers to enhance predictive power. However, individual polymorphisms in genes such as CALCA and RAMP1 showed inconsistent results. Although these genes were associated with migraine susceptibility in Christensen et al. (2016), their direct role in triptan response remained inconclusive.

### 3.4 Non‐Genetic Factors and Triptan Response

Non‐genetic factors also emerged as significant modulators of triptan response. Hormonal differences in women might contribute to variability in treatment efficacy (Gentile et al. 2010). This finding emphasizes the complex interplay between genetic predispositions and demographic or clinical characteristics influencing therapeutic outcomes.

### 3.5 Summary

The review highlighted consistent associations of variant rs4680 (Val158Met) in COMT and SLC6A4 STin2 VNTR with triptan response. Multi‐locus genetic models, such as the GRS, demonstrated promise in predicting treatment efficacy (Cargnin et al. 2015). However, serotonin receptor and dopaminergic gene variants showed variable findings, suggesting the need for further research to delineate their roles.

### 3.6 Overall Meta‐Analysis Findings

**FIGURE 2:**
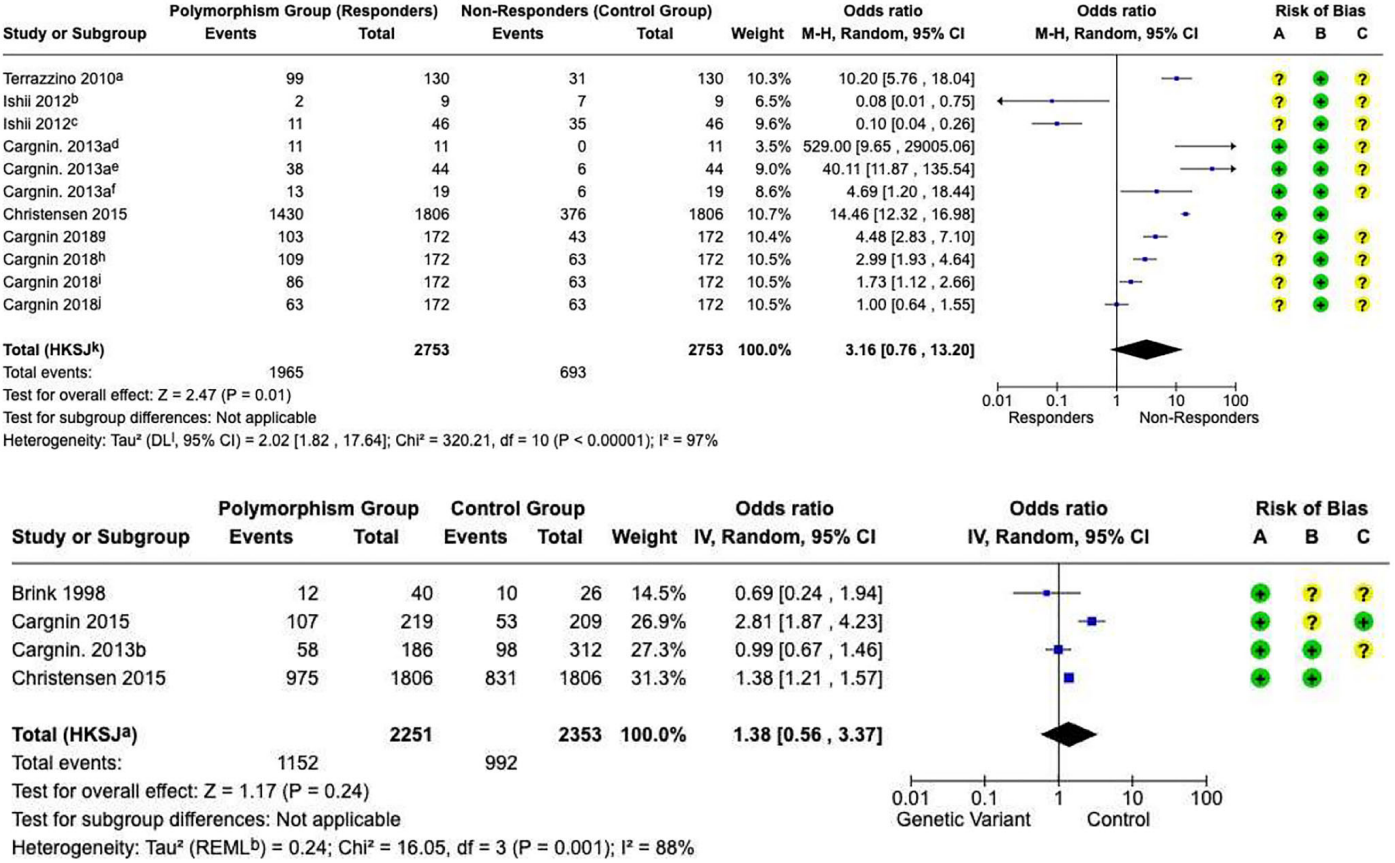
Comprehensive meta‐analysis of triptan response: overall effect and subgroup analysis. (A) The pooled odds ratio (OR) for triptan response across all included studies, differentiating between study designs (cohort vs. case‐control) to account for methodological variations. (B) Meta‐analysis stratified by genetic subgroups (neurotransmitter‐related, ion‐channel‐related, and migraine‐specific genes) to explore potential genetic contributions to triptan response heterogeneity.

A meta‐analysis was performed to enhance our understanding of the strength of the evidence presented in the research studies. Thus, we analyzed the overall effect size, heterogeneity, and potential moderators of genetic impact on triptan use in migraine treatment (Figure 2 and Table S4).

Since the studies had heterogeneity, the random‐effects model was used to combine the studies. The overall result was a pooled OR of 1.6642 (95% CI: 0.6414–4.3178, *p* = 0.2950), which did not show a statistically significant relationship between the genetic polymorphisms under investigation and triptan response. High heterogeneity was reported with *I* ^2^ = 94.1% (90.9%–96.2%), which indicates high variability between the included studies. The amount of heterogeneity was also estimated by *τ* ^2^ = 1.9851, and the corresponding *Q*‐statistic was 135.92 (df = 8, *p* < 0.0001).

### 3.7 Subgroup Analyses by Genetic Category

**FIGURE 3:**
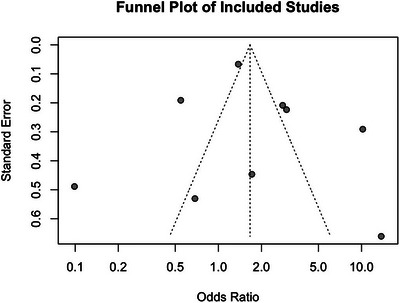
Funnel plot of the included studies. Funnel plot assessing potential publication bias. The degree of asymmetry in the plot provides insight into small‐study effects and the robustness of the meta‐analytic findings.

Since the studies were highly heterogeneous, the following subgroup analysis was carried out based on the genetic perspective into three categories (Figure 3 and Table S5):

A. Neurotransmitter‐related genes (e.g., SLC6A4, 5‐HT1B, COMT): This subgroup gave a total OR of 2.39, which suggests that there may be a relationship between these genes and triptan effectiveness.
B. Ion channel‐related genes (GRIA1, SCN1A): This group had a weaker correlation with the triptan response, because these genes are not directly linked to the pharmacodynamics of migraine.
C. Migraine‐related genes (CALCA, PRDM16): The migraine‐related genes showed a moderate association, which indicates that genes associated with migraine may also affect the response to triptans.

The analysis of Figure 3 suggests that neurotransmitter‐related genes (e.g., *SLC6A4, 5‐HT1B, and COMT*) exhibit the strongest association with triptan response (OR = 2.39, 95% CI [0.10–54.61]), supporting the role of serotonergic and dopaminergic pathways in triptan efficacy. Additionally, DRD2 (rs6275) was not included in subgroup analyses due to the limited availability of comparable data across studies. Migraine‐related genes (e.g., *CALCA, PRDM16*) show a moderate association (OR = 1.85, 95% CI [1.05–3.24]), suggesting a genetic influence on triptan response beyond pain pathways. Ion channel‐related genes (e.g., *GRIA1, SCN1A*) display a weaker association (OR = 0.90, 95% CI [0.30–2.74]), indicating that, while ion channels modulate neuronal excitability, their direct role in triptan pharmacodynamics may be limited. The results highlight the need for further investigation into the pharmacogenetic determinants of triptan response.

### 3.8 Sensitivity Analysis

**FIGURE 4:**
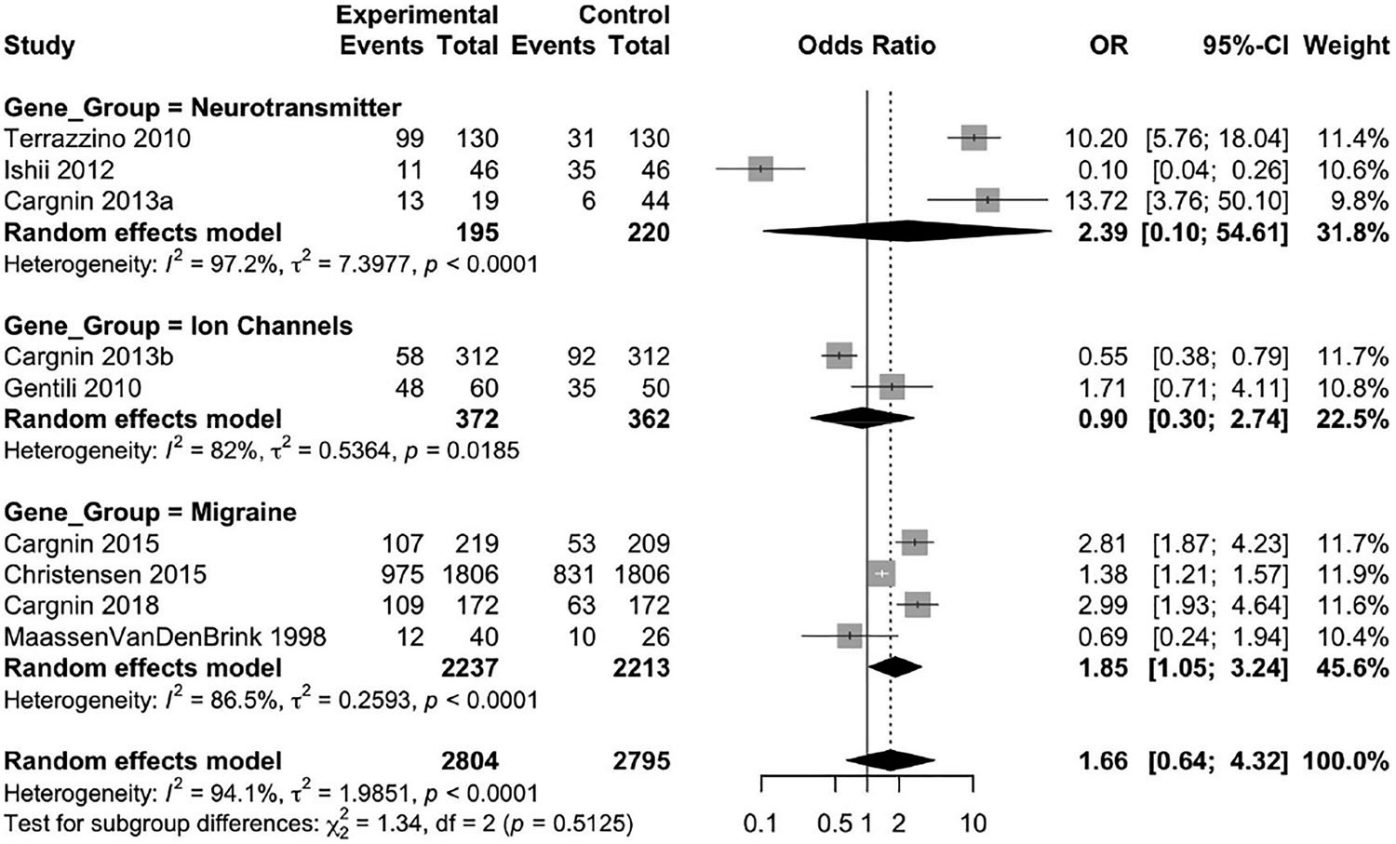
Subgroup analysis—forest plot by genetic category. This forest plot illustrates the results of the subgroup meta‐analysis, categorizing studies based on genetic function: neurotransmitter‐related genes, ion channel‐related genes, and migraine‐related genes. The pooled odds ratios (OR) and 95% confidence intervals (CI) are displayed for each subgroup, with the corresponding *I* ^2^, indicating heterogeneity.

**FIGURE 5:**
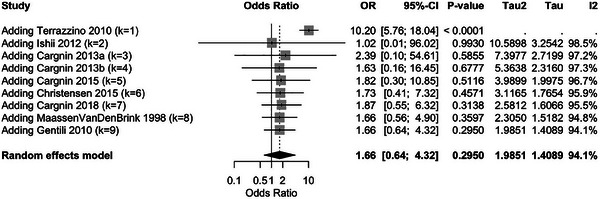
Sensitivity analysis—leave‐one‐out meta‐analysis. This forest plot illustrates the results of the leave‐one‐out sensitivity analysis, where each study was sequentially removed to assess its impact on the overall effect size.

The leave‐one‐out (Figures 4 and 5 and Table S6) analysis was performed to determine the stability of the meta‐analysis outcomes. The outcome of the analysis revealed that no study greatly affected the overall effect size, as the ORs did not change much when each study was excluded individually. The OR values were 0.27 (Terrazzino et al. 2010) and 0.83 (Ishii et al. 2012), indicating that the overall results were not based on any dataset; that is, no single study disproportionately influenced the meta‐analysis results. This supports the robustness and reliability of the pooled effect estimate.

### 3.9 Meta‐Regression for Moderator Analysis

To determine whether certain factors may lead to the heterogeneity of the results, a meta‐regression was carried out using two moderators: the year of publication and the total polymorphism count. The study further showed that the study year (*p* = 0.5818) and the total polymorphism count (*p* = 0.8821) did not differ in predicting triptan response variability. These results indicate that other factors, like differences in migraine diagnostic criteria and genetic methodologies, may cause the observed heterogeneity (Table S7).

### 3.10 Publication Bias Assessment

The funnel plot was used to evaluate the presence of publication bias. The plot's result showed moderate asymmetry, which may mean that minor study effects may affect the results. However, the Egger's test for funnel plot asymmetry did not reach the level of significance, thus indicating that publication bias was not a significant issue (Figure 2 and Table S3).

## 4 Discussion

The genetic influence on drug metabolism has been increasingly studied to develop predictive models that maximize drug response while minimizing adverse effects. Precision and personalized medicine have progressed significantly through pharmacogenetics and pharmacogenomics, with the most significant benefit being an individualized treatment, which enables greater therapeutic efficacy (Ihara et al. 2024). However, robust scientific evidence is still required to validate association studies in migraine treatment.

Our meta‐analysis found no statistically significant association between genetic variants and triptan response (OR = 1.66; 95% CI: 0.64–4.32; *p* = 0.295). First, the included studies were highly heterogeneous (*I* ^2^ = 94.1%), reflecting substantial variability in study populations, definitions of “negative response,” and the specific genes examined. Although the overall meta‐analysis did not show statistically significant associations, subgroup analyses revealed significant associations for SLC6A4, 5‐HT1B, and COMT polymorphisms. These findings support the hypothesis that genetic influence on triptan responsiveness may be context‐dependent.

A more detailed exploration of heterogeneity is essential. The high heterogeneity observed (*I* ^2^ = 94.1%) likely stems from differences in genetic testing methods (e.g., PCR vs. sequencing), study designs, population characteristics (e.g., ethnicity, age), and inconsistent clinical definitions of “nonresponse,” ranging from no pain relief to partial thresholds. Variations in triptan type, dosage, and timing further contributed. Although gene‐based subgrouping helped, residual variability remained, underscoring the need for methodological harmonization in future pharmacogenetic migraine studies. The polygenic nature of migraine suggests that aggregating multiple variants (e.g., through GRSs) may offer more predictive value than single polymorphism analyses.

Despite adopting formal methods to assess publication bias, this meta‐analysis identified moderate funnel plot asymmetry, suggesting potential small‐study effects that may inflate overall effect estimates. The lack of published negative or neutral findings undermines the evidence base's representativeness, limiting the generalizability of our conclusions. To address this, future studies should prioritize integrating grey literature and employing corrective methods like Trim and Fill to account for missing data. Given these issues, the non‐significant overall result should be interpreted cautiously: it does not refute genetic influence. Still, it shows that no single marker is currently a reliable standalone predictor. Moving forward, methodological transparency and broader evidence synthesis will be critical to advancing reliable, personalized migraine therapies.

Although gepants and ditans have broadened therapeutic options, clinical guidelines and real‐world practice continue to position triptans as the first‐line therapy for most acute attacks, balancing efficacy, long clinical experience, and access (Singh et al. 2020)

Genetic factors contribute to the variability in triptan response. Meta‐analysis indicated associations between SLC6A4, 5‐HT1B, and COMT polymorphisms and treatment outcomes. Despite high heterogeneity (*I* ^2^ = 94.1%), subgroup analyses identified neurotransmitter‐related genes as potential predictors (OR = 2.39, 95% CI [0.10–54.61]). These findings suggest a pharmacogenetic basis for triptan efficacy, supporting the exploration of genotype‐informed strategies in migraine therapy.

Methodological inconsistencies and small sample sizes, except in the study by Cargnin et al. (2019), limit the generalizability of findings. Larger prospective studies are necessary to validate these associations and enhance clinical applicability. Most studies employed candidate gene approaches focusing on specific polymorphisms. The availability of genomic data now facilitates comprehensive evaluations through genome‐wide association studies (GWAS) and Polygenic Risk Scores (PRS), assessing the cumulative impact of multiple genetic variants on drug response (Stel et al. 2021; Moola et al. 2015)

### 4.1 PRS and Combined Genetic Analyses

The current approach for migraine pharmacogenetics uses PRS and combined effect analyses (Grangeon et al. 2023; Cargnin et al. 2014; Kogelman et al. 2023; Ren et al. 2024) applied GWAS and PRS methods and found no SNPs significantly associated with triptan response after multiple testing correction. However, rs6724624 in the TRPM8 gene showed a nominal association, with the G allele linked to inconsistent response. They also proposed a GRS‐2 combining rs6724624 (TRPM8) and rs1024905 (FGF6), which significantly improved the prediction of consistent triptan response even after adjusting for triptan type Christensen et al. 2016 (Christensen et al. 2016) reported an association between the rs2651899 variant in the PRDM16 gene and improved triptan response, particularly in migraine with aura patients. Higher genetic risk burden correlated with better treatment outcomes.

### 4.2 Mendelian Randomization and Environmental Effects

Mendelian randomization analyses allow testing hypotheses regarding genetic versus environmental influences (Ren et al. 2024). Since genetic polymorphisms are gene variations that do not necessarily impair expression, they can occur in coding and non‐coding regions, affecting protein function (Christensen et al. 2016). These polymorphisms influence triptan treatment response, yet metabolic changes are also significant. Kogelman et al. (2023) demonstrated that alterations in GNAI1 and VIPR2 modulate molecular pathways, such as cAMP regulation, directly influencing sumatriptan efficacy. Therefore, Mendelian randomization studies can help separate environmental influences from genetic effects.

### 4.3 Candidate Genes for Triptan Metabolism

#### 4.3.1 Catechol‐*O*‐Methyltransferase

The COMT gene encodes an enzyme that degrades catecholamines and modulates pain and stress responses. The rs4680 (Val158Met) variant reduces enzyme activity by up to fourfold, increasing catecholamine levels and pain sensitivity in migraine (Cargnin et al., 2013). While not conclusively linked to migraine or menstrual migraine risk (Liao et al. 2017; Sutherland et al. 2017), it has been associated with depression and anxiety in chronic migraine (Fernández‐De‐Las‐Peñas et al. 2019). This variant is particularly relevant for frovatriptan response, with Met/Met carriers showing poorer outcomes (Cargnin et al. 2015). Other COMT variants, such as rs6269 and rs4818, also influence triptan efficacy (Terrazzino et al. 2010).

### 4.4 RAMP1 and CALCA Genes in CGRP Pathways

The RAMP1 gene encodes a protein crucial for CGRP receptor activation. Although the rs7590387 polymorphism has been linked to reduced migraine risk (Christensen et al. 2016), meta‐analyses have not confirmed this (Cargnin et al. 2015; Yasam et al. 2023), suggesting its role in medication‐overuse headache (MOH). The CALCA gene encodes CGRP, implicated in migraine pathophysiology through vasodilation and pain (Cargnin et al. 2019). Variants such as rs3781719 and rs3754701 have been associated with migraine and MOH (Cargnin et al. 2015; Terrazzino et al. 2010). The interaction between CALCA and RAMP1 highlights their functional relevance (Christensen et al. 2016), though epigenetic modifications also affect CGRP‐targeting therapy response (Fila et al. 2022; Carvalho et al. 2022)

Due to the absence of a definitive genetic biomarker, triptan use should remain clinically guided. Clinical features, comorbidities, and patient preferences remain central to treatment decisions, as no variant can yet pre‐identify non‐responders. If one triptan fails, alternatives like other triptans, NSAIDs, gepants, or ditans can be considered, since cross‐response varies (Cargnin et al. 2019). Our findings reinforce that, under current evidence, routine pharmacogenetic screening for triptan response is not justified in managing migraine. However, the observation that approximately 20 to 30 percent of patients present suboptimal responses to triptans (Lipton et al. 2010) supports the need for research exploring predictive factors related to treatment efficacy.

Subgroup analyses revealed that polymorphisms in neurotransmitter‐related genes, notably SLC6A4, 5‐HT1B, and COMT, exhibited a stronger association with triptan efficacy (pooled OR = 2.4), suggesting that variations in serotonergic and dopaminergic pathways may influence treatment response (Cargnin et al. 2019). In contrast, variants in ion channel‐related genes like GRIA1 and SCN1A showed weaker associations, indicating a potentially indirect role in acute treatment outcomes.

Moderate associations were observed for migraine susceptibility genes, including CALCA and PRDM16 (pooled OR = 1.8), implying that genetic predisposition may also affect pharmacological efficacy. However, these findings lacked definitive significance, highlighting the need for replication studies with larger, standardized cohorts.

Specific polymorphisms, such as COMT Val158Met (rs4680) and the STin2 VNTR in SLC6A4, have consistently correlated with triptan responsiveness across multiple studies (Christensen et al. 2016). The combination of these variants and the 5‐HTTLPR promoter polymorphism in SLC6A4 is recognized to affect neurotransmitter inactivation and is regarded as a biologically credible moderator of drug effects. Despite this, the evidence remains inconclusive, as replication across diverse demographic segments is inconsistent. This deficiency in reproducibility underscores the imperative for expansive, multi‐ethnic pharmacogenomic research, as well as more nuanced analyses of gene–gene and gene–environment interactions. For instance, hormonal status may modulate both migraine occurrence and medication response, indicating that variables such as estrogen levels could interact with genetic profiles to influence triptan efficacy, and epigenetic factors should also be considered (Lipton et al. 2010).

### 4.5 Implications

Early investigations into serotonin receptor variants (e.g., HTR1B G861C, T‐261G) and genes such as 5‐HTTLPR, MAOA‐VNTR, ESR1, MTHFR, ACE, TNF‐β, and GNB3 did not yield consistent associations with migraine or triptan response (MaassenVanDenBrink et al. 1998; Ishii et al. 2012). Our meta‐analysis confirmed these findings, showing no significant effect of these polymorphisms.

Although some studies suggested potential associations for CYP1A2*1F (rs762551) and MAOA uVNTR, our results did not support these and appear limited to specific cohorts without broader reproducibility (Gentile et al. 2010). These inconsistencies reinforce the need for larger, well‐powered studies to clarify the roles of these variants and validate their relevance to clinical outcomes.

Combined analyses and PRS are feasible approaches to assessing the genetic component of response to triptans. However, to increase clinical relevance, future research must focus on refining these models and integrating genetic predictors into treatment decisions through prospective studies and multi‐omics approaches.

### 4.6 Perspectives for Predictive Modeling and Precision Therapy

Integrating genetic, clinical, and biomarker data is essential for developing effective predictive models for migraine therapy. Personalized treatment strategies are advancing, and statistical and machine‐learning approaches have shown promise in predicting outcomes. However, systematic reviews (Chen et al. 2025) highlight that many models suffer from bias and lack external validation, limiting clinical applicability.

Prospective data collection is paramount for advancing model quality. Current models predominantly rely on retrospective analyses; prospective cohorts that capture treatment response predictors will yield superior evidence. Such data will ensure predictors like genetic profiles are documented before treatment, thus minimizing bias.

To reflect the complexity of migraines, models must encompass a diverse range of predictors. This incorporates genetic indicators, societal elements, features of headaches, accompanying conditions, and imaging findings, all of which can be examined via machine learning to discover detailed connections.

To ensure generalizability, models must undergo external validation in independent cohorts. Adherence to TRIPOD guidelines and tools like PROBAST is necessary to minimize bias and improve model calibration (Chen et al. 2025). Furthermore, clinical implementation demands user‐friendly, interpretable tools, ideally integrated into electronic health systems.

This paradigm shift toward individualized treatment using polygenic modeling may reduce the trial‐and‐error approach in migraine management and improve therapeutic outcomes.

### 4.7 Strengths, Limitations, and Future Directions

This study employed a rigorous methodological approach, applying strict inclusion criteria to ensure high‐quality evidence and minimize bias. The systematic review and meta‐analysis provided valuable insights into the pharmacogenetic determinants of triptan response, and the importance of SLC6A4, 5‐HT1B, and COMT polymorphisms was identified. Nevertheless, some drawbacks of this study should be mentioned.

One key limitation was the high heterogeneity observed (*I* ^2^ = 94.1%) (results section: overall meta‐analysis findings), likely due to differences in study design, sample size, and genotyping methodologies. Furthermore, most included studies were done in European and Asian populations, making it difficult to generalize the results to other ethnic groups. Additionally, most of the studies included were small, affecting the statistical power and making it challenging to produce robust results. Other factors were using different triptans, various dosing schedules, and different outcome measures. The critical gap that has not been addressed is the absence of replication in different cohorts. It stresses the necessity of conducting larger and well‐powered studies to confirm these findings before they can be implemented clinically.

Prospective trials and multi‐omics approaches, including genomic, transcriptomic, and metabolomic data to improve predictive models, could enhance the translational impact of future research. Expanding biomarker discovery and integrating more diverse populations is also essential because a personalized medicine framework for migraine treatment will require equitable and effective treatment strategies.

## 5 Conclusions

The overall analysis of our included articles shows high heterogeneity. Despite this variability in the data, we found more substantial evidence of the association between SLC6A4, 5‐HT1B, and COMT polymorphism with the response to Triptans when a subgroup analysis of genes with correlated functions was carried out. In contrast, *CALCA* and *PRDM16* polymorphisms showed moderate evidence, and GRIA1 and *SCN1A* showed weaker evidence to explain nonresponsive migraine patients to Triptan treatment. Combined analyses and polygenic risk scoring present viable methods for evaluating genetic influences on triptan efficacy. More studies using this approach could bring new insights to this area.

## Author Contributions

**Victoria Gomes Andreata**: investigation, conceptualization, methodology, visualization, formal analysis, software, writing – original draft, writing – review and editing. **Ruan Pablo Duarte Freitas**: investigation, methodology, visualization, formal analysis, software, writing – original draft, writing – review and editing. **Jéssica da Silva Nascimento**: investigation, conceptualization, methodology, visualization, writing – original draft. **Patrícia Sodré Araújo**: methodology, visualization, data curation. **Felipe Eustáquio dos Santos Guedes**: investigation, visualization. **João Vítor Benjamim Pires**: investigation, methodology, visualization, writing – original draft. **Narel Moita Carneiro Nogueira Falcão**: investigation, methodology, visualization. **Natasha da Silva Leitão**: investigation, methodology, visualization, data curation. **Alcylene Carla de Jesus dos Santos**: investigation, visualization. **Ana Patrícia Pascoal Queiroz**: investigation, methodology, visualization, writing – original draft. **Astria Ferrão Dias Gonzales**: investigation, methodology, validation, visualization. **Maica Matos Leão**: investigation, methodology, visualization, writing – original draft. **Emília Katiane Embiruçu de Araújo Leão**: investigation, visualization, data curation. **Juliana Côrtes Freitas**: investigation, methodology, validation, visualization, data curation, writing – original draft, writing – review and editing. **Acássia Benjamim Leal Pires**: investigation, conceptualization, methodology, validation, visualization, data curation, writing – review and editing, supervision, project administration.

## Conflicts of Interest

The authors declare no conflicts of interest.

## Peer Review

The peer review history for this article is available at https://publons.com/publon/10.1002/brb3.70967.

## Supporting information




**Supplementary Material**: brb370967‐sup‐0001‐SuppMat.docx

## Data Availability

The data that supports the findings of this study are available in the Supporting Information of this article.
