# Correction to “Genetic Basis of the Negative Response to the Use of Triptans for the Treatment of Migraine—A Systematic Review and Meta‐Analysis”

**DOI:** 10.1002/brb3.71067

**Published:** 2025-12-10

Andreata, V. G., R. P. D. Freitas, J. da S. Nascimento, et al. 2025. “Genetic Basis of the Negative Response to the Use of Triptans for the Treatment of Migraine—A Systematic Review and Meta‐Analysis.” *Brain and Behavior* 15, no. 10: e70967. https://doi.org/10.1002/brb3.70967

In the published version, the following funding information was missing:

Funding: The authors were partially supported by the Coordenação de Aperfeiçoamento de PessoaI de Nível Superior (CAPES), Finance Code 88881.015383/2024‐01, under Process number 1466/2025.

We apologize for this error.

