# Supplementary material for: Genetic Basis of the Negative Response to the Use of Triptans for the Treatment of Migraine—A Systematic Review and Meta‐Analysis
Source: Brain Behav. 2025 Oct 21;15(10):e70967. doi: 10.1002/brb3.70967 (PMC12541135; doi:10.1002/brb3.70967)
Supplement: Supplementary file 1 — Supplementary Material: brb370967‐sup‐0001‐SuppMat.docx [file BRB3-15-e70967-s001.docx]

**|11|Table of content**

**List of Tables**

Table S1. Preferred Reporting Items for Systematic Reviews and Meta-Analyses (PRISMA) checklist………...2

Table S2. Search strategy used in different databases …………………………………………………………....4

Table S3. Risk of Bias Assessment of Individual Studies………………………………………………...…........6

Table S4. Meta-Analysis Results…………………..…....…………...…………………………………................7

Table S5. Subgroup Analysis by Genetic Category……………………...………………………..........................7

Table S6. Sensitivity Analysis - Leave-One-Out Approach……………...…………………...…..........................8

Table S7. Meta-Regression Results…………………….……………...…………………….…..........................10

Table S8. Study Populations and Triptan Use……………….………...………………………...........................10

**Statistical analsysis**

Advanced Statistical Analysis and Genetic Insights into Triptan Response: Supplementary Findings………....13

**Table S1. Preferred Reporting Items for Systematic Reviews and Meta-Analyses (PRISMA) checklist^1^**

| **Section and Topic** | **Item #** | **Checklist item** | **Reported on page number** |
| --- | --- | --- | --- |
| **TITLE** | | |  |
| Title | 1 | Identify the report as a systematic review. | Page 1 |
| **ABSTRACT** | | |  |
| Abstract | 2 | See the PRISMA 2020 for Abstracts checklist. | Page 2 |
| **INTRODUCTION** | | |  |
| Rationale | 3 | Describe the rationale for the review in the context of existing knowledge. | Page 3 |
| Objectives | 4 | Provide an explicit statement of the objective(s) or question(s) the review addresses. | Page 3 |
| **METHODS** | | |  |
| Eligibility criteria | 5 | Specify the inclusion and exclusion criteria for the review and how studies were grouped for the syntheses. | Page 4 |
| Information sources | 6 | Specify all databases, registers, websites, organisations, reference lists and other sources searched or consulted to identify studies. Specify the date when each source was last searched or consulted. | Page 5 |
| Search strategy | 7 | Present the full search strategies for all databases, registers and websites, including any filters and limits used. | Supplementary Table S2 |
| Selection process | 8 | Specify the methods used to decide whether a study met the inclusion criteria of the review, including how many reviewers screened each record and each report retrieved, whether they worked independently, and if applicable, details of automation tools used in the process. | Page 6 |
| Data collection process | 9 | Specify the methods used to collect data from reports, including how many reviewers collected data from each report, whether they worked independently, any processes for obtaining or confirming data from study investigators, and if applicable, details of automation tools used in the process. | Page 6 |
| Data items | 10a | List and define all outcomes for which data were sought. Specify whether all results that were compatible with each outcome domain in each study were sought (e.g. for all measures, time points, analyses), and if not, the methods used to decide which results to collect. | Page 7 |
|  | 10b | List and define all other variables for which data were sought (e.g. participant and intervention characteristics, funding sources). Describe any assumptions made about any missing or unclear information. | Page 7 |
| Study risk of bias assessment | 11 | Specify the methods used to assess risk of bias in the included studies, including details of the tool(s) used, how many reviewers assessed each study and whether they worked independently, and if applicable, details of automation tools used in the process. | Page 8 (Table S3) |
| Effect measures | 12 | Specify for each outcome the effect measure(s) (e.g. risk ratio, mean difference) used in the synthesis or presentation of results. | Page 9 |
| Synthesis methods | 13a | Describe the processes used to decide which studies were eligible for each synthesis (e.g. tabulating the study intervention characteristics and comparing against the planned groups for each synthesis (item #5)). | Page 9 |
|  | 13b | Describe any methods required to prepare the data for presentation or synthesis, such as handling of missing summary statistics, or data conversions. | Page 9 |
|  | 13c | Describe any methods used to tabulate or visually display results of individual studies and syntheses. | Page 9 |
|  | 13d | Describe any methods used to synthesize results and provide a rationale for the choice(s). If meta-analysis was performed, describe the model(s), method(s) to identify the presence and extent of statistical heterogeneity, and software package(s) used. | Page 10 |
|  | 13e | Describe any methods used to explore possible causes of heterogeneity among study results (e.g. subgroup analysis, meta-regression). | Page 10 |
|  | 13f | Describe any sensitivity analyses conducted to assess robustness of the synthesized results. | Page 10 |
| Reporting bias assessment | 14 | Describe any methods used to assess risk of bias due to missing results in a synthesis (arising from reporting biases). | Not Found |
| Certainty assessment | 15 | Describe any methods used to assess certainty (or confidence) in the body of evidence for an outcome. | Not Found |
| **RESULTS** | | |  |
| Study selection | 16a | Describe the results of the search and selection process, from the number of records identified in the search to the number of studies included in the review, ideally using a flow diagram. | Page 11 (Figure 1) |
|  | 16b | Cite studies that might appear to meet the inclusion criteria, but which were excluded, and explain why they were excluded. | Page 12 |
| Study characteristics | 17 | Cite each included study and present its characteristics. | Page 12 (Table 1) |
| Risk of bias in studies | 18 | Present assessments of risk of bias for each included study. | Page 13 (Table S3) |
| Results of individual studies | 19 | For all outcomes, present, for each study: (a) summary statistics for each group (where appropriate) and (b) an effect estimates and its precision (e.g. confidence/credible interval), ideally using structured tables or plots. | Page 14 |
| Results of syntheses | 20a | For each synthesis, briefly summarise the characteristics and risk of bias among contributing studies. | Page 14 |
|  | 20b | Present results of all statistical syntheses conducted. If meta-analysis was done, present for each the summary estimate and its precision (e.g. confidence/credible interval) and measures of statistical heterogeneity. If comparing groups, describe the direction of the effect. | Page 15 (Figure 2) |
|  | 20c | Present results of all investigations of possible causes of heterogeneity among study results. | Page 16 |
|  | 20d | Present results of all sensitivity analyses conducted to assess the robustness of the synthesized results. | Page 17 (Figure 3) |
| Reporting biases | 21 | Present assessments of risk of bias due to missing results (arising from reporting biases) for each synthesis assessed. | Not Found |
| Certainty of evidence | 22 | Present assessments of certainty (or confidence) in the body of evidence for each outcome assessed. | Not Found |
| **DISCUSSION** | | |  |
| Discussion | 23a | Provide a general interpretation of the results in the context of other evidence. | Page 18 |
|  | 23b | Discuss any limitations of the evidence included in the review. | Page 18 |
|  | 23c | Discuss any limitations of the review processes used. | Page 18 |
|  | 23d | Discuss implications of the results for practice, policy, and future research. | Page 19 |
| **OTHER INFORMATION** | | |  |
| Registration and protocol | 24a | Provide registration information for the review, including register name and registration number, or state that the review was not registered. | Page 4 (PROSPERO ID: CRD2024598554) |
|  | 24b | Indicate where the review protocol can be accessed, or state that a protocol was not prepared. | Not Found |
|  | 24c | Describe and explain any amendments to information provided at registration or in the protocol. | Not Found |
| Support | 25 | Describe sources of financial or non-financial support for the review, and the role of the funders or sponsors in the review. | Not Found |
| Competing interests | 26 | Declare any competing interests of review authors. | Not Found |
| Availability of data, code and other materials | 27 | Report which of the following are publicly available and where they can be found: template data collection forms; data extracted from included studies; data used for all analyses; analytic code; any other materials used in the review. | Not Found |

**Table S2. Search Strategy Across Databases for the Identification of Genetic Predictors of Triptan Response in Migraine**

| **Search strategy Pubmed** | | | |  | **Results** |
| --- | --- | --- | --- | --- | --- |
|  | **Concept 1 - Migraine** | **Concept 2 - Genetic Factors** | **Concept 3 - Triptans** |  | **#1 AND #2 AND #3:** |
| **Mesh term** | **"Migraine Disorders"[Mesh] OR "Migraine with Aura"[Mesh] OR "Migraine without Aura"[Mesh]** | **"Pharmacogenomic Variants"[Mesh] OR "Genome-Wide Association Study"[Mesh] OR "DNA Copy Number Variations"[Mesh] OR "Cytochrome P-450 Enzyme System"[Mesh] OR "Polymorphism, Genetic"[Mesh] OR "Polymorphism, Single Nucleotide"[Mesh] OR "Genetic Association Studies"[Mesh] OR "Genetic Risk Score"[Mesh] OR "Genetic Variation"[Mesh] OR "Genes"[Mesh] OR "Alleles"[Mesh]** | **"Serotonin 5-HT1 Receptor Agonists"[Mesh] OR "Sumatriptan"[Mesh] OR "Tryptamines"[Mesh]** |  | **592 results** |
| **Keyword** | **“Migraine with Auras” [tw] OR “Familial Hemiplegic Migraine” [tw] OR “Complicated Migraine” [tw] OR “Classic Migraine” tw] OR “Acute Confusional Migraine” [tw] OR “Migraine Hemicrania” [tw] OR “Migraine” [tw] OR “Headache, Migraine” [tw] OR “Common migraine” [tw]** | **“GWA” [tw] OR “GWA Study” [tw] OR “Genome Wide Association Scan” [tw] OR “Copy Number Polymorphism” [tw] OR “CNV” [tw] OR “CNP” [tw] OR “Genetic Polymorphism” [tw] OR “Single Nucleotide Polymorphism” [tw] OR “SNPs” [tw] OR “Gene Discovery” [tw] OR “Genotype Phenotype Correlations” [tw] OR "CYP2D6" [tw] OR "CYP3A4" [tw] OR "CYP1A2" [tw] OR "MAO-A" [tw] OR "CYP2C9" [tw] OR "CYP2C19" [tw] OR "Cytochrome P450" [tw] "genetics" [tw] OR "gene" [tw] OR "genetic factors" [tw] OR "variants" [tw] OR "variant" [tw] OR "gene polymorphism" [tw] OR "gene polymorphisms" [tw] OR "Risk Score, Genetic" [tw] OR "indel mutation" [tw] OR "sequence deletion" [tw] OR "insertion mutation" [tw] OR "mutation" [tw] OR "gene duplication" [tw] OR "DNA Copy Number Variant" [tw] OR "Oxidase, Monoamine" [tw]** | **“Triptans” OR “Triptan” OR “zolmitriptan” [Supplementary Concept] OR “naratriptan” [Supplementary Concept] OR “xaliproden” [Supplementary Concept] OR “eletriptan” [Supplementary Concept] OR “rizatriptan” [Supplementary Concept] OR “zolmitriptan” [Supplementary Concept] OR “almotriptan [Supplementary Concept]** |  |  |
| **Total** | **48,536 results** | **3,857,339 results** | **105,166 results** |  |  |
| **Search strategy Cochrane** | | | |  | **Results** |
|  | **Concept 1 - Migraine** | **Concept 2 - Genetic Factors** | **Concept 3 - Triptans** |  | **#1 AND #2 AND #3:** |
| **Terms** | **“Transtornos de Enxaqueca” OR “Trastornos Migrañosos” OR “Migraine Disorders” OR "Migraine Disorders" OR "Migraine with Aura" OR "Migraine without Aura" OR “Migraine with Auras” OR “Familial Hemiplegic Migraine” OR “Complicated Migraine” OR “Classic Migraine” OR “Acute Confusional Migraine” OR “Migraine Hemicrania” OR “Migraine”** | **“Variantes Farmacogenômicos” OR “Variantes Farmacogenómicas” OR “Pharmacogenomic Variants” OR "Pharmacogenomic Variants" OR "Genome-Wide Association Study" OR "DNA Copy Number Variations" OR "Cytochrome P-450 Enzyme System" OR "Polymorphism, Genetic" OR "Polymorphism, Single Nucleotide" OR "Genetic Association Studies" OR "Genetic Risk Score" OR "Genetic Variation" OR "Genes" OR "Alleles" OR “GWA” OR “GWA Study” OR “Genome Wide Association Scan” OR “Copy Number Polymorphism” OR “CNV” OR “CNP” OR “Genetic Polymorphism” OR “Single Nucleotide Polymorphism” OR “SNPs” OR “Gene Discovery” OR “Genotype Phenotype Correlations” OR "CYP2D6" OR "CYP3A4" OR "CYP1A2" OR "MAO-A" OR "CYP2C9" OR "CYP2C19" OR "Cytochrome P450"** | **“Triptaminas” OR “Triptaminas” OR “Tryptamines” OR "Serotonin 5-HT1 Receptor Agonists" OR "Sumatriptan" OR "Tryptamines" OR “zolmitriptan” OR “naratriptan” OR “xaliproden” OR “eletriptan” OR “rizatriptan” OR “zolmitriptan” OR “almotriptan”** |  | **58 results** |
| **Total** |  | | |  |  |
| **Search strategy Web of Science** | | | |  | **Results** |
|  | **Concept 1 - Migraine** | **Concept 2 - Genetic Factors** | **Concept 3 - Triptans** |  | **#1 AND #2 AND #3:** |
| **Terms** | **ALL=(Migraine Disorders OR Migraine OR (Headache, Migraine)** | **ALL=(genetics OR gene OR genes OR genetic OR genetic factors OR variants OR variant OR Alleles OR Genetic Variation OR DNA Copy Number Variant OR Pharmacogenomic Variant OR Pharmacogenomic Variants OR (Polymorphism, Genetic) OR (Polymorphism, Single Nucleotide) OR (gene polymorphism) OR (gene polymorphisms) OR (Copy Number Polymorphism) OR (Genetic Polymorphism) OR (Genotype Phenotype Correlations) OR (Polygenic Risk Score) OR (genetic risk score) OR (Risk Score, Genetic) OR (Risk Score, Polygenic)** | **ALL=(Serotonin 5-HT1 Receptor Agonists) OR Sumatriptan OR Tryptamines OR Tryptamines OR zolmitriptan OR naratriptan OR xaliproden OR eletriptan OR rizatriptan OR zolmitriptan OR almotriptan OR Triptan OR Triptans)** |  | **771 results** |
| **Total** |  | | |  |  |

**Table S3. Risk of Bias Assessment of Individual Studies (NOS: Newcastle-Ottawa Scale)**

| **ARTICLES** | **DOMAINS** | **TOTAL** |
| --- | --- | --- |
| MassenVanDerBrink , 1998 | Selection: ★★★★ Comparability: ★ Outcome: ★ | 7 |
| Gentile et al., 2010 | Selection: ★★ Comparability: ★★ Outcome: ★ | 5 |
| Terrazzino et al., 2010 | Selection: ★★ Comparability: ★★ Outcome: ★ | 9 |
| Ishii et al., 2012 | Selection: ★★ Comparability: ★★ Outcome: ★ | 5 |
| Cargnin et al., 2013a | Selection: ★★★★ Comparability: ★★ Outcome: ★ | 7 |
| Cargnin et al., 2013b | Selection: ★★★ Comparability: ★★ Outcome: ★ | 6 |
| Cargnin et al., 2015 | Selection: ★★★ Comparability: ★ Outcome: ★★★ | 7 |
| Christensen et al., 2015 | Selection: ★★★★ Comparability: ★★ Outcome: - | 6 |
| Cargnin et al., 2018 | Selection: ★★ Comparability: ★★ Outcome: ★ | 5 |

#### Table S4: Meta-Analysis Results

## Subtitle:

S4 presents the overall meta-analysis results, summarizing the pooled Odds Ratio (OR) and 95% Confidence Interval (CI) for all included studies. The analysis evaluates the association between genetic variants and triptan response using a random-effects model. The pooled OR was 1.66 (95% CI: 0.64–4.32, p = 0.2950), indicating a non-significant association across the studies.

High heterogeneity was observed (I² = 94.1%), suggesting substantial variability among the studies. The Q-test for heterogeneity was statistically significant (Q = 135.92, df = 8, p < 0.0001), further supporting the presence of variability and the necessity for subgroup analyses. The restricted maximum-likelihood (REML) estimator was applied to model this heterogeneity.

This table underscores the overall effect estimate while highlighting the considerable heterogeneity, justifying the need for further sensitivity and subgroup analyses.

| **Metric** | **Value** |
| --- | --- |
| **Number of studies** | 9 |
| **Number of observations** | 5599 (o.e = 2804, o.c = 2795) |
| **Number of events** | 2588 |
| **Random effects model OR** | 1.6642 |
| **95%-CI** | [0.6414; 4.3178] |
| **Z value** | 1.05 |
| **P-value** | 0.295 |
| **Tau²** | 1.9851 [0.8256; 8.2023] |
| **Tau** | 1.4089 [0.9086; 2.8640] |
| **I²** | 94.1% [90.9%; 96.2%] |
| **H** | 4.12 [3.31; 5.14] |
| **Test of heterogeneity (Q)** | 135.92 |
| **Degrees of freedom (d.f.)** | 8 |
| **P-value (heterogeneity)** | < 0.0001 |

#### Table S5: Subgroup Analysis by Genetic Category

## Subtitle: Table S5 examines heterogeneity by categorizing genetic variants into three biologically relevant subgroups. Neurotransmitter-related genes (SLC6A4, 5-HT1B, COMT) showed a stronger association with triptan response (pooled OR = 2.39, 95% CI: 0.10–54.61) but with high heterogeneity (I² = 97.2%).

## Ion channel-related genes (GRIA1, SCN1A) had a weaker association (pooled OR = 0.90, 95% CI: 0.30–2.74) and lower heterogeneity (I² = 82%). Migraine-related genes (CALCA, PRDM16) demonstrated a moderate association (pooled OR = 1.85, 95% CI: 1.05–3.24) with substantial heterogeneity (I² = 86.5%).

## These findings suggest neurotransmitter genes may strongly influence triptan efficacy, while ion channel and migraine-related genes contribute differently.

| **Genetic Category** | **Genes Included** | **Pooled OR (95%-CI)** | **I² (%)** |
| --- | --- | --- | --- |
| **Neurotransmitter-Related Genes** | SLC6A4, 5-HT1B, COMT | 2.39 [1.14–5.01] | 89.3 |
| **Ion Channel-Related Genes** | GRIA1, SCN1A | 1.21 [0.71–2.06] | 82.7 |
| **Migraine-Specific Genes** | CALCA, PRDM16, CYP1A2 | 1.78 [0.96–3.31] | 91.2 |

#### Table S6: Sensitivity Analysis - Leave-One-Out Approach

## Subtitle: Each study was omitted in turn and the meta-analysis repeated for the leave-one-out sensitivity analysis. The OR estimates were quite variable following exclusions, and ranged from 0.27 to 0.83, but the overall trend was still consistent. The largest drop in OR was seen on exclusion of Terrazzino 2010 at 0.27, indicating strong influence on the overall result. Some effects, for example, Ishii 2012 (OR = 0.83), had relatively small impact on the final estimate. This table shows that the results are not presented based on one study only and therefore support the reliability of the pooled effect estimate.

| **Study** | **Leave-One-Out OR** |
| --- | --- |
| **Terrazzino 2010** | 0.2739012 |
| **Ishii 2012** | 0.8314757 |
| **Cargnin 2013a** | 0.2849716 |
| **Cargnin 2013b** | 0.6568476 |
| **Cargnin 2015** | 0.4409585 |
| **Christensen 2015** | 0.5352063 |
| **Cargnin 2018** | 0.4329598 |
| **MaassenVanDenBrink 1998** | 0.6136031 |
| **Gentili 2010** | 0.5062112 |

#### Table S7: Meta-Regression Results

## Subtitle: Table S7 presents meta-regression analyses evaluating whether publication year or total polymorphism count contributed to study heterogeneity. Study year (p = 0.5818) was not a significant moderator, indicating temporal differences did not impact effect size variability. Similarly, total polymorphism count (p = 0.8821) showed no significant association with OR estimates, suggesting sample size or variant frequency did not explain the heterogeneity.

## These results suggest that other factors, such as migraine classification, genetic methods, or population differences, may account for the observed heterogeneity.

| **Metric** | **Value** |
| --- | --- |
| **logLik** | -13.2116 |
| **deviance** | 26.4233 |
| **AIC** | 32.4233 |
| **BIC** | 32.261 |
| **AICc** | 40.4233 |
| **Tau² (Residual Heterogeneity)** | 2.3212 (SE = 1.3264) |
| **Tau** | 1.5235 |
| **I² (Unaccounted Variability)** | 0.9632 |
| **H² (Sampling Variability)** | 27.19 |
| **R² (Heterogeneity Accounted)** | 0 |
| **Test for Residual Heterogeneity (QE)** | 130.9634 (df = 7), p < .0001 |
| **Test of Moderators (QM)** | 0.0220 (df = 1), p = 0.8821 |

##

| **Model Results** | **Estimate** | **SE** | **z-value** | **p-value** | **CI Lower** | **CI Upper** |
| --- | --- | --- | --- | --- | --- | --- |
| **Intercept** | 0.5561 | 0.6094 | 0.9124 | 0.3615 | -0.6384 | 1.7505 |
| **Total Polymorphism** | -0.0001 | 0.001 | -0.1483 | 0.8821 | -0.002 | 0.0017 |

##

## Table S8: Study Populations and Triptan Use

## Subtitle: Summary of genetic variants analyzed in the included studies and their associations with triptan response. This table highlights the genetic polymorphisms evaluated, categorized by gene function, and their reported influence on treatment outcomes

| **Study** | **Genes** | **Genetic Variants** | **Results** |
| --- | --- | --- | --- |
| **MassenVanDerBrink et al., 1998** | HTR1B (5-HT1B receptor gene) | - G861C  - T-261G | No significant association. |
| **Gentile et al., 2010** | MAOA  CYP1A2  GNB3 | - uVNTR (3R/4R variants)  - CYP1A2 1F (rs762551)  - C825T (rs5443) | Significant association of MAOA uVNTR with triptan response (dependent on repeat number). CYP1A2 -163A (rs762551) linked to excessive triptan use. |
| **Terrazzino et al., 2010** | SLC6A4  GNB3  DRD2 | - 5HTTLPR  - STin2 VNTR  - C825T  - TaqI A  - NcoI | STin2 VNTR (SLC6A4) linked to inconsistent triptan response. Other polymorphisms showed no effect. |
| **Ishii et al., 2012** | - 5-HTT (SLC6A4)  - 5-HT2A Receptor  - 5-HT1B Receptor  - MAOA  - MTHFR  - ACE  - ESR1  - DRD2  - TNF-β  - GNB3 | - 5-HTTLPR (NG_011747)  - 5-HTTVNTR (NG_011747)  - 5-HT2A | Only STin2 VNTR (SLC6A4) polymorphism was associated with inconsistent triptan response. |
| **Cargnin et al., 2013a** | COMT | - Val158Met (rs4680) (Genotypes: Val/Val, Val/Met, Met/Met) | Carriers of COMT 158Met allele had an increased risk of poor response to frovatriptan compared to Val/Val homozygotes. |
| **Cargnin et al., 2013b** | GRIA1 | - rs548294  - rs2195450 | No significant difference in triptan response between migraine patients and controls. |
| **Cargnin et al., 2015** | CALCA  RAMP1 | - rs3781719 (T > C)  - rs3754701 (T > A)  - rs7590387 (C > G) | No direct effect on triptan response. However, rs7590387 (RAMP1) was linked to a ›lower risk of medication-overuse headache. |
| **Christensen et al., 2015** | PRDM16  LRP1  TRPM8  TSPAN2  MEF2D  ASTN2  TGFBR2  AJAP1  C7orf10  PHACTR1  MMP16 | SNP Variants Evaluated:  - rs10915437  - rs11172113  - rs12134493  - rs13208321 (Surrogate for rs11759769)  - rs2274316  - rs2651899  - rs4379368  - rs6478241  - rs6790925  - rs7577262 (Surrogate for rs6741751)  - rs9349379  - rs10504861 | PRDM16 rs2651899 significantly associated with triptan efficacy. Other SNPs showed variable associations with triptan or preventive drugs. |
| **Cargnin et al., 2018** | TRPM8  FGF6  LRP1  ASTN2  TSPAN2  PHACTR1 | - TRPM8: rs6726424  - FGF6: rs1024905  - LRP1: rs11172113, rs6478241  - ASTN2: rs2651899  - TSPAN2: rs12134493  - PHACTR1: rs9349379 | Genetic risk score predicted triptan efficacy in migraine without aura. PRDM16 variants were associated with improved treatment response. |

###

**1.Supplementary Statistical Analysis Section:**

Additional details on the statistical methodologies employed to investigate the genetic basis of triptan response in migraine patients are provided in this section. Tabular presentation showcases subgroup analysis results, sensitivity testing outputs, and meta-regression findings to support the main manuscript conclusions.

**1.1. Meta-Analysis and Heterogeneity Assessment:**

A random-effects model estimated the overall effect size because of significant study-to-study variability. The substantial heterogeneity between studies (I² = 94.1%) clearly required further exploration through subgroup analysis. The Mantel-Haenszel procedure provided the pooled Odds Ratios (OR) with 95% Confidence Intervals (CI) using fixed categorisation. The Q-test demonstrated important heterogeneity among studies which was highly significant (Q = 135.92, df = 8, p < 0.0001).

*See Table 2 and S4.*

**2. Analysis of Subgroups by Genetic Category**

The studies were divided into genetic function groups because actual heterogeneity was substantial to identify which gene categories predicted triptan response. The subgroup analysis investigated whether genetic modifications in neurotransmission or ion channel function and migraine predisposition affected the probability of response to triptan.

*See Figure 3*

**2.1. Neurotransmitter Related Genes**

The group consists of genes which play important roles in serotonergic and dopaminergic transmission since these pathways are central to migraine disease and triptan drug action.

Genes analyzed:

The gene which codes for the serotonin transporter SLC6A4 controls the reabsorption of serotonin in the brain. 5-HT1B is the gene for the serotonin receptor which serves as the principal focus of triptan action. The COMT gene controls the breakdown process of dopamine through its catechol-O-methyltransferase function.

Findings:

The combined Odds Ratio (OR) result was 2.39 which indicated a possible link between these genes and triptan effectiveness.

Results indicate that the serotonergic system plays a role in how triptans work.

The high degree of heterogeneity indicates that other factors including receptor binding and balance mechanisms between neurotransmitters play a role.

*See Figure 3 and S5.*

**2.2. Genes associated with Ion Channels**

These genes influence neuronal excitability, pain transmission and cortical spreading depression (CSD), an important migraine pathway. Genes analyzed: GRIA1 (gene encoding a glutamate receptor, for excitatory neurotransmission). SCN1A (sodium channel gene, neuronal excitability modifier). Findings: The pooled OR was 0.90, meaning a weaker association with triptan response than with neurotransmitter related genes. Although these genes are linked with migraine risk, their effect on triptan pharmacodynamics is thought to be indirect. Further work is required to explore if certain polymorphisms are linked with triptan resistance.

*See Figure 3 and S5*

**2.3. Genes related to Migraine**

These genes are linked to migraine predisposition and not to the direct pharmacokinetics of triptans. Genes analyzed: CALCA (calcitonin gene related peptide, CGRP, one of the major migraine mediators). PRDM16 (a gene that controls neurovascular functions and has been linked to migraine genetics). Findings: The pooled OR was 1.85, indicating a moderate association of migraine specific genes with triptan response. The wide CIs show that this category has genetic variation which may produce opposite effects on triptan response. Supports the concept that genetic susceptibility to migraine affects the response to medication.

*See Figure 3 and S5*

**3. Sensitivity Analysis**

A leave-one-out analysis had the purpose of checking if some studies could have affected the results disproportionately. The work of each study was excluded and the meta-analysis was re-run to check on the stability of the results. The overall OR stayed within the range of 0.27 to 0.83, thus indicating that no study influenced the final pooled estimate significantly. The meta-analysis results suggest that there is stability of the results and that the findings are not due to a specific study.

*See Table S6 and Figure 4*

**4. Publication Bias Assessment**

A funnel plot was used to determine if publication bias existed in the data.

Small trial sizes with unfavourable results suggested moderate asymmetry indicating possible publication bias.

Due to the low number of included studies (k = 9), the power to examine publication bias is critical.

Egger’s test was not performed because of insufficient power.

*See Figure 2 and S3*

**5. Meta-Regression for Moderator Analysis**

To explore possible sources of heterogeneity, meta-regression was performed on two moderators:

*See Table S7*

**5.1. Study Year as a Moderator**

Rationale: To find out if the changes in genetic approaches or the use of diagnostic criteria over the years affected the response to triptans.

Findings:

The p-value was 0.5818, meaning that the study year did not systematically influence the OR estimates.

Presents the finding that changing genetic approaches and classification systems did not cause the observed relationships.

*See Table S7*

**5.2. Sample Size (Total Polymorphism Count) as a Moderator**

Rationale: To determine if small studies with high variance contaminated the results.

Findings:

The p-value was 0.8821, indicating that there was no significant impact of sample size on the pooled effect estimate.

Suggests that study power and precision were not the main causes of heterogeneity.

*See Table S7*

**6. Summary of Key Findings**

The strongest association with triptan response was observed in neurotransmitter-related genes (OR = 2.39), consistent with involvement of serotonin and dopamine pathways.

Ion channel-related genes had a weaker correlation (OR = 0.90), which suggests that these genes may be associated with triptan pharmacodynamics in a indirect way.

The genes specific to migraines presented a moderate association (OR = 1.85), which suggests that there could be a genetic component to how individuals respond to treatment.

Sensitivity analysis was performed to check the robustness of the results and no study was found to significantly change the effect size.

Meta-regression analysis revealed that study year and sample size did not account for the heterogeneity, thus other methodological factors might be responsible for the variability.

The result showed publication bias, but due to the small number of studies, it is difficult to interpret the result correctly.
